# Comparison of Gabapentin and Ketotifen in Treatment of Uremic Pruritus in Hemodialysis Patients

**DOI:** 10.12669/pjms.321.8547

**Published:** 2016

**Authors:** Saeid Amirkhanlou, Anna Rashedi, Jalal Taherian, Ali Akbar Hafezi, Sahar Parsaei

**Affiliations:** 1 Dr. Saeid Amirkhanlou, MD, Assistant Professor, Nephrologist, Department of Internal Medicine, Golestan University of Medical Sciences, Gorgan, Golestan, Iran; 2 Dr. Anna Rashedi, MD, Radiologist, Assistant Professor, Department of Internal Medicine, Golestan University of Medical Sciences, Gorgan, Golestan, Iran; 3 Dr. Jalal Taherian, MD, General Practitioner, Department of Internal Medicine, Golestan University of Medical Sciences, Gorgan, Golestan, Iran; 4 Dr. Ali Akbar Hafezi, MD, General Practitioner, Department of Internal Medicine, Golestan University of Medical Sciences, Gorgan, Golestan, Iran; 5 Sahar Parsaei, Medical Student, Department of Internal Medicine, Golestan University of Medical Sciences, Gorgan, Golestan, Iran

**Keywords:** Gabapentin, Ketotifen, Uremic Pruritus, Hemodialysis

## Abstract

**Objectives:**

Uremic pruritus is a common problem in hemodialysis patients. Several treatments have been used for decreasing itching in these patients. Gabapentin and ketotifen are two drugs used for treating uremic patients. The aim of this study was to compare gabapentin and ketotifen in treatment of uremic pruritus in hemodialysis patients.

**Methods:**

In this double-blind randomized clinical trial, 52 hemodialysis patients with uremic pruritus referred to 5azarTeaching Hospital in Gorgan in 2013 were studied. Patients were randomly assigned to two groups of 26 subjects (groups G and K). In group G, patients treated with gabapentin capsules 100 mg daily for 2 weeks, and in Group K, patients treated with ketotifen 1 mg twice daily for 2 weeks. Before and at the end of study, pruritus severity was determined based on Shiratori’s severity scores. Collected data were analyzed by SPSS-21 statistical software.

**Results:**

There was no significant different between two groups in the age and sex. After two weeks of treatment, severity of pruritus was significantly reduced in both groups (88.4% in group G vs. 76.9% in group K). Gabapentin compared with ketotifen had a better effect on improving itching in the age group of 30-60 years and in males. 5 patients (19.2%) in both groups suffered from drowsiness and dizziness, but no serious side effects were observed.

**Conclusions:**

The results showed that gabapentin and ketotifen significantly improved pruritus in hemodialysis patients, and no significant difference was observed between two groups.

## INTRODUCTION

Pruritus is one of the biggest problems in hemodialysis patients. Unfortunately, approximately 37-90% of the patients with ESRD suffer from itching.^1^,^2^ Pathophysiology of pruritus in patients with chronic renal failure (CRF) is unknown.^3^,^4^ Uremic pruritus usually appears before starting the treatment with hemodialysis and gradually progresses.^5^,^6^

Given the various causes of pruritus in hemodialysis patients, several treatments have been used and investigated.^7^,^8^ Few studies have been done on the effectiveness of gabapentin with antihistamines such ketotifen.^9^,^10^ In this study, the effects of gabapentin and ketotifen on improving uremic pruritus were compared.

## METHODS

This double-blind randomized clinical trial was conducted after approval from the Research Ethics Committee of Golestan University of Medical Sciences. Of 182 hemodialysis patients referred to 5 azar Teaching Hospital in Gorgan in 2013, 52 eligible patients with uremic pruritus were selected. All patients undergoing hemodialysis were similar in frequency and method (maximum duration and frequency of hemodialysis) and patients suffering from itchy skin condition (non-uremic pruritus) were excluded. Patients were randomly assigned to two groups of 26 subjects (groups G and K). In group G, patients treated with gabapentin capsules (Iran Daroo Pharmaceutical Co., Tehran, Iran) 100 mg daily for 2 weeks, and in Group K, patients treated with ketotifen (Abidi Pharmaceutical Co., Tehran, Iran) 1 mg twice daily for 2 weeks. The patients and drug distributors were not aware of the prescribed medications. For matching two groups, before beginning the study, serum calcium, phosphorus and hemoglobin were reached to above 8 mg/dL, less than 5 mg/dL and about 11 to 12 gr/dL, respectively, and the patient were controlled about anemia and hyperparathyroidism.

Before and at the end of study, pruritus severity were determined based on Shiratori’s severity scores (0= no itching, 1= minimal, 2= mild, 3= moderate and 4= severe itching).^11^ Clinical response to treatment was determined as follows: (1) Complete response (no itching or minimal itching after treatment), (2) Partial response (mild or moderate severity of itching after treatment) and (3) No response (severe pruritus after treatment). Two groups of patients were compared in terms of age, gender, response to treatment and medication side effects (dizziness, drowsiness, tachycardia and palpitations). Collected data were analyzed by SPSS-21 statistical software and chi-square (χ^2^) and t tests, Fisher’s exact test and Mann-Whitney U test.

## RESULTS

A total of 52 hemodialysis patients with uremic pruritus in two groups were treated with gabapentin and ketotifen. The mean age of patients in group G was 60.2±7.4 years, and in group K was 57.6±6.2 years, which was not statistically significant (P=0.127). In group G, 12 patients (46.2%) were males and 14 patients (53.8%) were females, and in group K, 13 patients (50%) were males and 13 patients (50%) were females, which was not significantly different (P=0.098).

**Fig.1:**
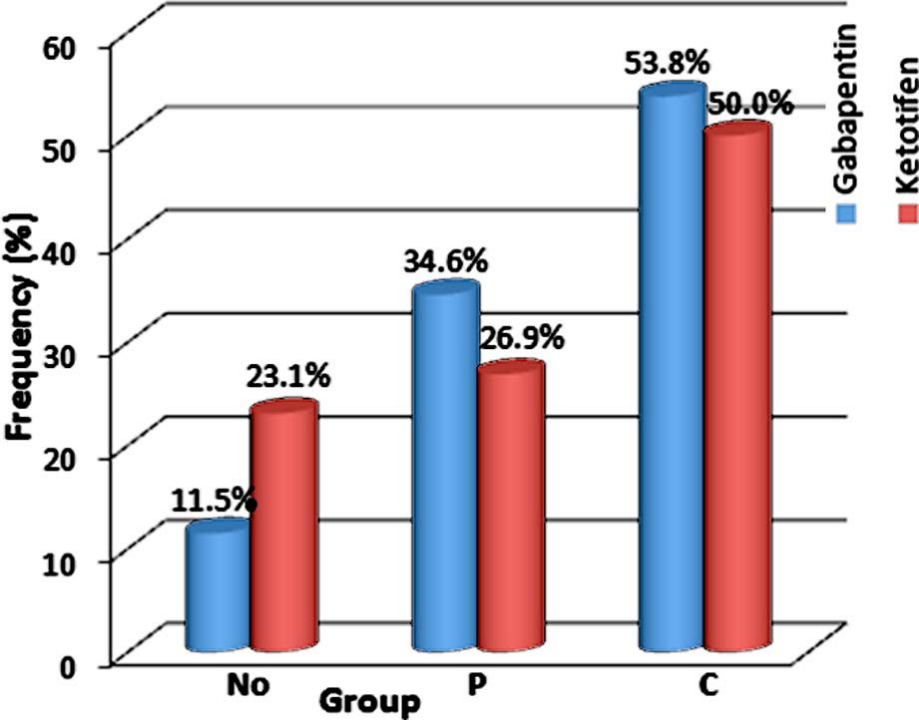
Comparison of the response to treatment with gabapentin and ketotifen in hemodialysis patients with uremic pruritus. No (no response), P (Partial response), and C (Complete response).

The results showed that of 52 patients, 49 patients (94.2%) reported itching during the day and 47 patients (90.4%) at night. In group G, 3 patients (11.5%) did not respond to treatment, 9 patients (34.6%) had a partial response and 14 patients (53.8%) had a complete response to treatment. In group K, 6 patients (23.1%) did not respond to treatment, 7 patients (26.9%) had a partial response and 13 patients (50.0%) had a complete response to treatment. Hence, there was no significant difference between two groups in terms of the response to treatment (P=0.481) (Fig.1).

Comparing the effects of gabapentin and ketotifen in the treatment of uremic pruritus by age showed that in the age group of 30-60 years, a significant difference was observed between two groups in terms of response to treatment (P= 0.008), but in other age groups, There were no significant differences (P>0.05) (Table-I).

**Table-I:** Comparison of the effects of gabapentin and ketotifen in the treatment of uremic pruritus in hemodialysis patients by age.

Age Groups (year)		Response to Treatment						Total		P value

		No		Partial		Complete				

		Frequency	Percent	Frequency	Percent	Frequency	Percent	Frequency	Percent	
Under 30	G*	1	1.9	0	0	2	3.8	3	5.8	0.659
	K**	2	3.8	1	1.9	3	5.8	6	11.5	
30-60	G	0	0	6	11.5	6	11.5	12	23.1	0.008
	K	3	5.8	2	3.8	6	11.5	11	19.2	
Upper 60	G	2	3.8	3	5.8	6	11.5	11	19.2	0.854
	K	1	1.9	4	7.7	4	7.7	9	17.3	

Comparing two treatment groups by sex showed that there was a significant difference in the males between two groups in terms of response to treatment (P= 0.011), but among the females, There was no significant difference (P=0.258) (Table-II).

**Table-II:** Comparison of the effects of gabapentin and ketotifen in the treatment of uremic pruritus in hemodialysis patients by sex.

Sex		Response to Treatment						Total		P value

		No		Partial		Complete				

		Frequency	Percent	Frequency	Percent	Frequency	Percent	Frequency	Percent
Male	G	1	1.9	4	7.7	8	15.4	13	25.0	0.011
	K	3	5.8	3	5.8	6	11.5	12	23.1	
Female	G	2	3.8	5	9.6	6	11.5	13	25.0	0.258
	K	3	5.8	4	7.7	7	13.5	14	26.9	

Assessment of the side effects in both treatment groups showed that in both groups, 4 patients (15.4%) experienced drowsiness and 1 patient (3.8%) had dizziness after taking gabapentin and Ketotifen, that there was no statistically significant difference (P=1.00) (Table-III). Also, no cases with palpitations and tachycardia were observed.

**Table-III:** Comparison of the side effects of gabapentin and ketotifen in hemodialysis patients with uremic pruritus.

Side Effect Drug	Drowsiness		Dizziness		No Side Effect		Total		P value

	Frequency	Percent	Frequency	Percent	Frequency	Percent	Frequency	Percent	
Gabapentin	4	7.7	1	1.9	21	40.4	26	50.0	1.00
Ketotifen	4	7.7	1	1.9	21	40.4	26	50.0	

## DISCUSSION

In this study, the effects of gabapentin and ketotifen on treating hemodialysis patients with uremic pruritus were studied over a two week period. The results showed that both groups of patients significantly responded to treatment though the response to treatment was greater in gabapentin group (88.4% vs 76.9%), but there was no significant difference between two groups. Also, in the age group of 30-60 years, a significant difference was found between two groups in response to treatment, so that patients of gabapentin group showed a better response to treatment. Moreover, in males, a significant difference was observed between two groups in terms of response to treatment, so that patients treated with gabapentin respond better to treatment. It seems that ketotifen is not superior to gabapentin in the treatment of uremic pruritus and patient response to treatment with gabapentin has been relatively better than ketotifen. But in terms of side effects, a small number of patients in both groups had drowsiness and dizziness and no palpitations and tachycardia were reported. Therefore, the use of these drugs in the treatment of uremic pruritus showed no serious side effects.

The studies done by other researchers had varying results. Marquez, et al. in their study about comparing the efficacy and safety of desloratidine (an antihistamine) versus gabapentin showed that pruritus was decreased with both treatments but There were no differences when comparing the final pruritus score with both drugs. Excessive sedation was common with gabapentin, while desloratadine was well tolerated.^12^ In the present study, gabapentin and ketotifen (an antihistamine) were significantly improved pruritus in hemodialysis patients, although the efficacy of gabapentin was slightly better than ketotifen but no significant difference was found between two groups in terms of the side effects. Rayner, et alby compared the effects of gabapentin and pregabalin in reducing pruritus. They concluded that gabapentin relieved itching in 66% of the patients, while pregabalin relieved itching in 81% of the patients. Also, 37% of patients suffered side effects from gabapentin.^9^ In the present study gabapentin improved pruritus in 88.4% of the patients. Finally, only 5 patients (19.2%) had drowsiness and dizziness, but none of the patients had serious side effects. Razeghi, et al. in their study on uremic pruritus refractory to antihistamines showed that gabapentin is an effective agent in treating uremic pruritus and no significant correlation was found between age and sex with gabapentin effect.^13^ This study also demonstrated the therapeutic effects of gabapentin in uremic patients, but in the age group of 30-60 years and the males, gabapentin relieved pruritus in hemodialysis patients more significant than ketotifen. Gunal, et alin their study showed that the severity of pruritus in patients treated with gabapentin significantly decreased more than control group. Also, no significant complications were observed. Therefore, gabapentin in the treatment of uremic pruritus appeared to be safe and effective.^14^ Vila, et al. in their review article showed that gabapentin has demonstrated efficacy in the treatment of multiple types of itch especially in treating patients with uremic patients who are unresponsive to standard therapies. All of the controlled studies consisted of 4 weeks of active treatment, and no patients discontinued gabapentin due to adverse events.^15^ In the study of Manenti, et al. all patients experienced a rapid subjective improvement in pruritus by gabapentin.^16^

There are very few studies on the effects of ketotifen compared gabapentin in ESRD patients, however, a number of studies have shown the beneficial effects of ketotifen in the treatment of uremic pruritus. In the study of Francos, et al. determined all uremic patients treated with ketotifen had significant reductions in pruritus at 8 weeks after treatment.^17^ Noshad&NazariKhanmiri in a study on comparison of gabapentin and antihistamins (hydroxyzine) in treatment of uremic pruritus showed that reducing the severity of pruritus in gabapentin group (group G) was significantly higher than hydroxyzine group (group A). Itching remained in 10% of the patients in group G versus 80% in group A. The side effects were 35% in group G versus 50% in group A.^10^ Khalili, et al. in their study about effect of three antihistamines on uremic pruritus in the patients with CRF showed that hydroxyzine (33%) and chlorpheniramine (20%) significantly reduced the severity of itching in uremic patients, but ketotifen (4.5%) was insignificantly decreased pruritus.^18^ But in the present study, ketotifen relieved itching in 76.9% of patients.

It is most likely that uraemic pruritus is a mixture of both neuropathic and neurogenic itch. Neuropathic itch can originate from damage of the nervous system located at any point along the afferent pathway. Because its pathophysiology of uremic pruritus is poorly understood, the treatment of uraemic pruritus remains mainly empirical.^14^ Among the various drugs used in the treatment of uremic pruritus, gabapentin and ketotifen were investigated in this study. Although its mechanism of action is not clear, gabapentin appears to have an effect on voltage-dependent calcium-ion channels. By inhibiting neuronal calcium influx, it may interrupt the series of events that perhaps lead to the pruritic sensation in uraemia.^19^ On the other hand, it seems that histamine in combination with other factors plays an important role in the pathogenesis of pruritus,^20^,^21^ although this finding has not been established in some studies.^22^,^23^ Ketotifen is also an antiserotonergic agent and mast cell stabilizer, have been effective in controlling pruritus of some patients.^17^

## CONCLUSION

The results of this study showed that gabapentin and ketotifen significantly improved pruritus in hemodialysis patients. Although the effect of gabapentin was better than ketotifen but no significant difference was observed between two groups. Also, in the age group of 30-60 years and in males, gabapentin was significantly more effective than ketotifen. Taking these drugs had few side effects in patients, therefore, this study indicated the efficacy and safety of gabapentin and ketotifen in the treatment of uremic pruritus, although further studies are needed to confirm the results.
